# Reduced-Dose Pazopanib as First-Line Therapy in Metastatic Renal Cell Carcinoma: Real-World Outcomes and Subsequent Treatment Patterns from a Resource-Constrained Setting in South India

**DOI:** 10.15586/jkc.v13i3.495

**Published:** 2026-09-08

**Authors:** Arun Krishnan M.P., Nandini Devi R., Praveen K. Shenoy, Shoaib Nawaz, Abhilash Menon

**Affiliations:** 1 Department of Clinical Hematology and Medical Oncology, Malabar Cancer Center, Thalassery, Kannur, Kerala, India

**Keywords:** LMIC, metastatic renal cell carcinoma, reduced dose pazopanib

## Abstract

Immune checkpoint inhibitor-based combinations are the current standard first-line treatment for metastatic renal cell carcinoma (mRCC). However, tyrosine kinase inhibitors remain the predominant first-line therapy in many low- and middle-income countries because of limited affordability and access to immunotherapy. Dose reduction of tyrosine kinase inhibitors is frequently practiced in real-world settings to improve tolerability and treatment adherence. However, data on outcomes with reduced-dose pazopanib and subsequent treatment patterns remain limited. This retrospective study evaluated the effectiveness and safety of reduced-dose pazopanib (400 mg daily) as first-line systemic therapy in anti-VEGF treatment-naive patients with mRCC who were unable to access immunotherapy. Patients aged ≥18 years with histologically confirmed RCC who received reduced-dose pazopanib between January 2016 and December 2023 were included. Data on response rates, toxicities, and subsequent treatment lines were collected from hospital records. Survival outcomes were estimated using the Kaplan–Meier method. Eighty-four patients received reduced-dose pazopanib during the study period. The median age was 57.5 years (range, 19–76 years). Most patients were male (79%) and had clear-cell histology (79.7%). Sixty-six per cent of patients presented with de novo metastatic disease, while 52% belonged to the International Metastatic RCC Database Consortium (IMDC) intermediate-risk group. Nearly half of the cohort (45.1%) had an Eastern Cooperative Oncology Group (ECOG) performance status ≥2. Nephrectomy was performed in 52 patients (61.9%); of these, radical nephrectomy in 29 (58%) and cytoreductive nephrectomy in 23 (42%). Clinical benefit at the first response assessment was observed in 69 patients (82.1%). At a median follow-up of 41.3 months, median progression-free survival and overall survival were 8.9 months (95% CI, 6.7–11.0 months) and 28.6 months (95% CI, 19.0–38.1 months), respectively. The most common adverse events were skin and hair depigmentation (35.7%), diarrhea (25%), hand–foot syndrome (21.4%), and hypertension (14.3%). Grade 3–4 adverse events occurred in 5.9% of patients, and treatment discontinuation due to toxicity occurred in 2.4%. No treatment-related deaths were reported. Reduced-dose pazopanib appears to be a feasible first-line treatment option for anti-VEGF treatment-naïve patients with mRCC who are unable to access immunotherapy, providing clinically meaningful efficacy with a favorable toxicity profile.

## Introduction

Renal cell carcinoma (RCC) accounts for 2–3% of all malignancies. Clear cell carcinoma is the most common histologic subtype (1, 2). In India, nearly 33% of cases are diagnosed as de novo metastatic disease, and approximately one-third of patients with localized disease who undergo nephrectomy will subsequently progress to stage 4 disease (3, 4).

Systemic therapy is the main modality of treatment for advanced clear cell RCC. Over the past two decades, the standard of care in metastatic clear cell RCC evolved from tyrosine kinase inhibitor (TKI) monotherapy to immune checkpoint inhibitor (ICI)–TKI combination therapy (5). But in low- and middle-income countries (LMICs), where accessibility to and affordability of immunotherapy are limited, most patients receive TKI monotherapy (6, 7). This approach has been endorsed as a practically feasible option by the PEARL-INDIA consensus (7). Pazopanib is one of the most commonly used TKIs. The Phase 3 trial by Sternberg et al. established pazopanib 800 mg once daily as a treatment option for metastatic treatment-naïve kidney cancer, demonstrating a significant objective response rate (ORR) of approximately 32% and progression-free survival (PFS) benefit (8). The COMPRAZ trial confirmed similar efficacy and favorable quality-of-life profiles for pazopanib compared with sunitinib (9). Further findings from a meta-analysis by Deng et al. demonstrated that pazopanib was a cost-effective alternative to sunitinib, with similar antitumor efficacy (10).

Although pazopanib demonstrated significant efficacy in pivotal registration trials, treatment-related toxicities were common. In the phase III trial by Sternberg et al., frequent adverse events included diarrhea (52%), hypertension (40%), hair color changes (38%), nausea (26%), and elevated alanine aminotransferase levels (18%), resulting in dose reductions or treatment interruptions in a substantial proportion of patients. Similar toxicity patterns were observed in the COMPARZ trial, particularly among Asian patients, supporting the need for treatment strategies that improve tolerability while maintaining clinical efficacy (8, 11). Tolerance of standard-dose TKIs in Indian patients is poor. Data from an Indian kidney cancer registry reported that one-fourth of patients with mRCC treated with first-line TKIs or mTOR inhibitors required dose reductions from standard dosing during treatment (12). In large prospective real-world studies, standard-dose pazopanib (800 mg) also frequently required dose reductions because of treatment-related toxicities. In the APOLON study cohort, one-third of patients discontinued pazopanib because of adverse events, and nearly half (47%) required dose reduction during treatment (13).

Few studies suggest that pazopanib dose optimization may improve tolerability without compromising efficacy. Tanaka et al. explored the feasibility of optimizing pazopanib dosing based on plasma concentrations. This Japanese study demonstrated that a pazopanib dose of 400 mg provided a therapeutically effective plasma concentration and was associated with a favorable and well-tolerated toxicity profile (14). A retrospective study from Georgia by Stitt et al. showed that a reduced initial starting dose of a TKI in patients with mRCC improved tolerability without adversely affecting clinical outcomes (15). Akatsuka et al. from Japan reported that a starting dose of 400 mg of pazopanib did not adversely affect clinical outcomes and was associated with improved tolerability (16). In a multicenter study by the PRINCIPAL study group, 9.6% of patients who received 400 mg of pazopanib had a longer duration of drug exposure than those who received the standard dose (6.9 vs 9.6 months) (17). A post hoc analysis of the COMPARZ study published in 2019 reported that patients who underwent dose reductions following adverse events received higher cumulative doses and had longer treatment exposure than those who continued treatment at the standard dose (18). These factors were associated with improved ORRs and survival outcomes in the reduced-dose pazopanib cohort. Collectively, these findings demonstrate that dose modifications of pazopanib can be safely implemented without compromising efficacy or long-term outcomes (19).

However, reduced-dose pazopanib is not currently considered standard first-line therapy for mRCC because evidence regarding this approach remains conflicting across different populations. A retrospective study by Grassi et al. from Italy concluded that reduced-dose pazopanib significantly reduced the ORR compared with the standard dose of 800 mg (44% vs 19%) (20). Furthermore, although 26.6 and 14.7% of patients received reduced-dose pazopanib (400 mg) in the PAZOREAL and PARACHUTE studies, respectively, evidence specifically evaluating outcomes with an exclusive 400 mg starting dose remains limited (21, 22). These findings from real-world studies suggest that dose reduction is a frequently adopted strategy across diverse healthcare settings. Given that dose tailoring is a common practice in India to improve treatment adherence and tolerability, further real-world evidence on the effectiveness of reduced-dose pazopanib is needed.

Sequencing of treatment options after first-line TKI progression in patients who cannot afford immunotherapy remains an unresolved problem. Although numerous evidence-based subsequent-line treatment options are available, the majority are not covered by reimbursement schemes. This often results in the use of less effective treatment options in subsequent lines of therapy in resource-constrained settings (23, 24).

This study evaluated the effectiveness and safety of reduced-dose pazopanib (400 mg) as first-line therapy in anti-VEGF treatment-naïve patients with metastatic renal cell carcinoma (mRCC). Furthermore, the study describes the patterns of subsequent lines of therapy after reduced-dose pazopanib in a resource-constrained setting in South India.

## Methods

This was a retrospective observational study conducted in the Department of Medical Oncology at a tertiary cancer center in South India. We reviewed the medical records of patients diagnosed with RCC who received reduced-dose pazopanib (400 mg) as first-line systemic therapy between January 2016 and December 2023. The study received approval from the Institutional Review Board in December 2025 (IRB No. 1616/IRB-SRC/13/MCC/13-12-2025/4), and a waiver of informed consent was obtained because of the retrospective nature of the study.

The objectives of the study were to assess response rates, toxicities, and survival outcomes associated with reduced-dose pazopanib as first-line systemic therapy and to document patterns of subsequent lines of treatment received by this patient cohort.

### Study population

Patients with biopsy-proven mRCC who received pazopanib 400 mg as first-line systemic therapy were included in the study. Patients with incomplete records regarding pazopanib dosing details and those who had received prior TKIs or immunotherapy in the adjuvant setting were excluded. Patients underwent monthly clinical assessments to evaluate treatment tolerance, toxicity, and performance status. Routine laboratory investigations, including complete blood counts and renal and hepatic biochemical parameters, were performed monthly during the first 3 months and subsequently every 2 months. Thyroid function tests were assessed approximately every 3 months. Additional investigations were performed when clinically indicated, particularly in the presence of symptoms or laboratory abnormalities suggestive of treatment-related toxicity. The first radiological response assessment was generally performed 3–4 months after initiation of pazopanib using RECIST version 1.1, with subsequent imaging performed based on clinical suspicion of disease progression or other clinical indications.

### Data collection

Unique Health Identifier numbers of patients with histologically confirmed RCC patients were obtained from the hospital cancer registry, and individual patient records were screened to identify those meeting the study eligibility criteria. Data on clinicopathologic characteristics, response rates, toxicities, and subsequent lines of treatment were extracted from hospital case records and entered into IBM SPSS Statistics version 29 software.

### Statistical analysis

Statistical analyses were performed using IBM SPSS Statistics version 29.0. Categorical variables were expressed as frequencies and percentages. Survival curves were estimated using the Kaplan–Meier method.

## Operational Definitions

- Overall survival (OS) was calculated from the date of initiation of pazopanib treatment until death from any cause or the date of the last follow-up.
- Progression-free survival (PFS) was calculated from the date of initiation of pazopanib treatment until the date of disease progression or death from any cause.
- Treatment response was assessed radiologically according to the Response Evaluation Criteria in Solid Tumors (RECIST), version 1.1.
- Tumor response was categorized as complete response (CR), partial response (PR), stable disease (SD), or progressive disease (PD) based on changes in measurable target and non-target lesions.
- Overall response rate (ORR) was calculated as the sum of CR and PR.
- Clinical benefit rate (CBR) was calculated as the proportion of patients achieving CR, PR, or SD.

## Results

After screening the records of 305 patients with RCC, 84 patients with Stage IV disease who received reduced-dose pazopanib as first-line systemic therapy and met the study eligibility criteria were identified. The baseline clinicodemographic characteristics of the patient cohort are presented in Table 1. The median age at presentation was 57.5 years, with a male predominance (78.5%). Regarding treatment modalities received before initiation of pazopanib, 52 (61.9%) patients underwent nephrectomy. Among these 52 patients, radical nephrectomy was performed in 29 (58%), while cytoreductive nephrectomy was performed in 23 (42%). Metastasis-directed treatment before initiation of pazopanib consisted of palliative radiation to metastatic sites in 17 patients (20%) and radiofrequency ablation in 1 patient (1.2%).

**Table 1:** Baseline characteristics (n = 84).

Characteristics	Patient numbers (%)
**Age**	
≤ 39 years	3 (3.6)
40–49 years	16 (19.0)
50–59 years	32 (38.1)
60–69 years	26 (31.0)
≥ 70 years	7 (8.3)
Median age	57.5 years (19–76 years)
**Gender**	
Male	66 (78.5)
Female	18 (21.4)
**ECOG performance status**	
0	2 (2.4)
1	44 (52.4)
2	27 (32.1)
3	11 (13.1)
**Laterality**	
Right	41 (48.8)
Left	42 (50.0)
Bilateral	1 (1.2)
**Histological subtype**	
Clear cell	67 (79.7)
Unclassified (No subtype characterized)	17 (20.2)
**Stage at time of diagnosis**	
I	3 (3.8)
II	9 (10.7)
III	17 (20.2)
IV	55 (65.5)
**IMDC risk status**	
Favorable	21 (25.0)
Intermediate	44 (52.4)
Poor	11 (13.1)
Not available	8 (9.5)
**Site of metastasis**	
Lung	70%
Bone	30%
Brain	5%
Liver	17%
More than two sites of metastasis	36%

ECOG, Eastern Cooperative Oncology Group; IMDC, International Metastatic Renal Cell Carcinoma Database Consortium.

### Response rates and clinical outcomes to reduced dose pazopanib

**Figure 1:**
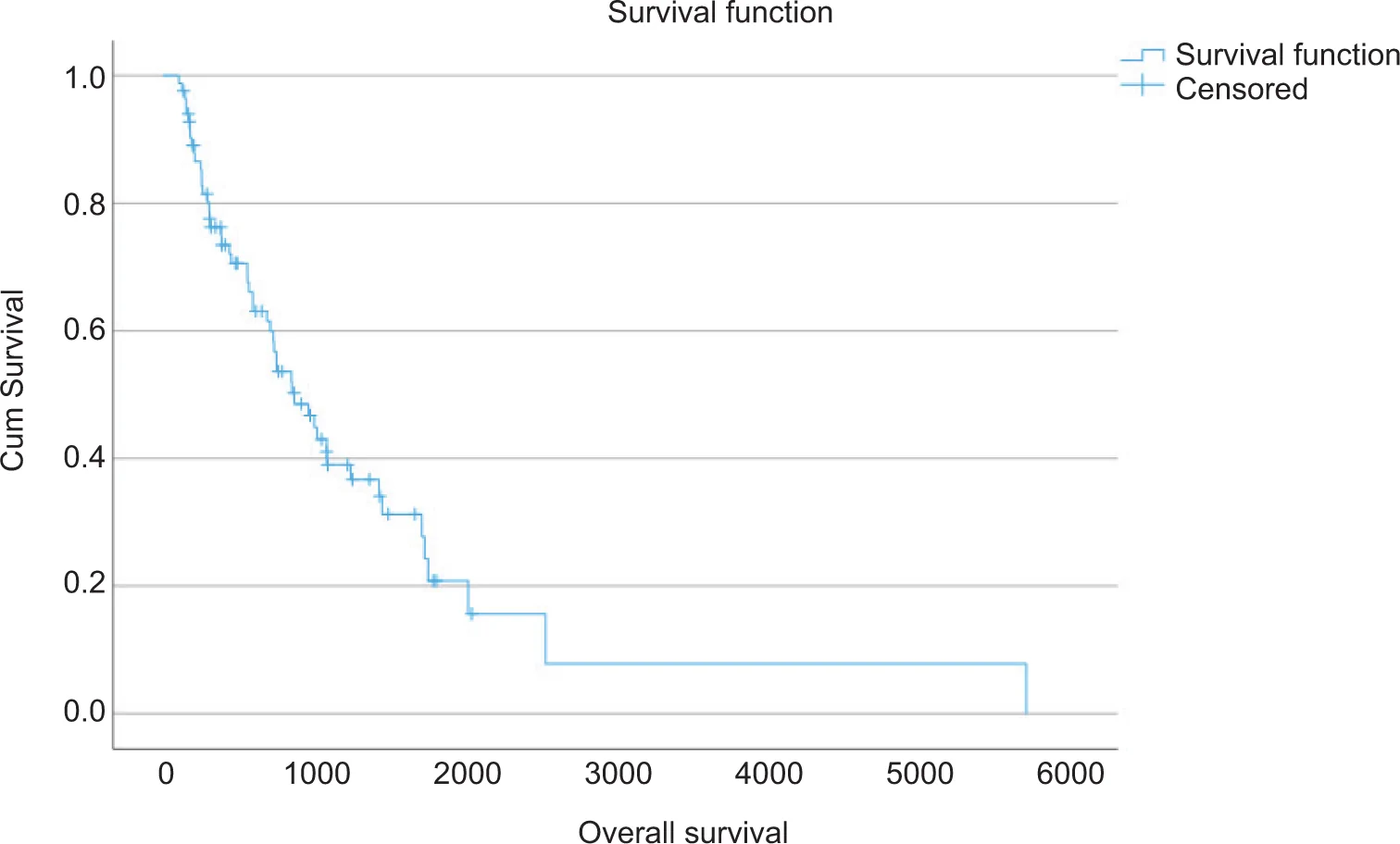
Overall survival (OS) in days.

**Figure 2:**
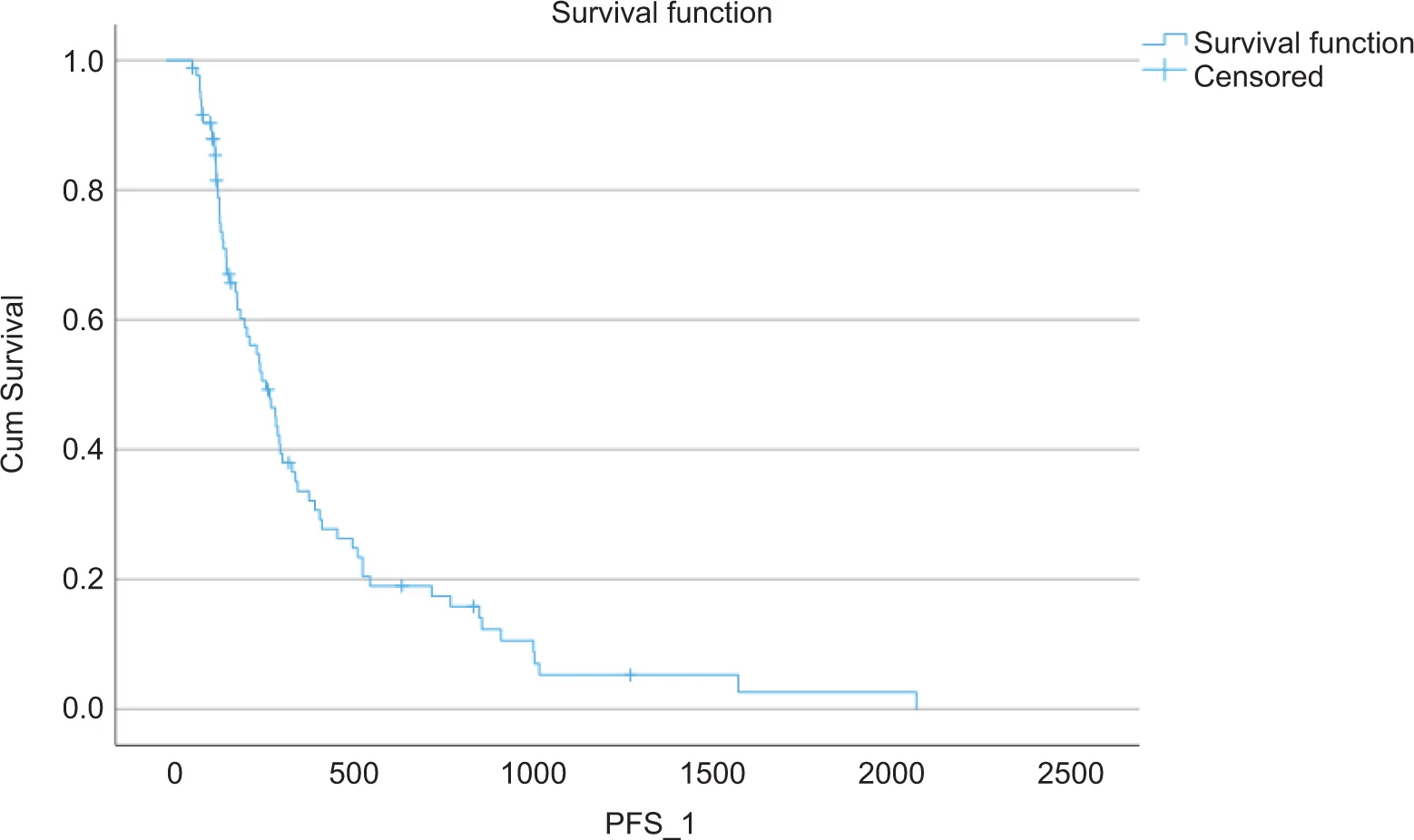
Progression-free survival (PFS_1) in days.

All patients (84) were initiated on pazopanib at 400 mg, and tumor response assessment imaging was performed in 82 patients. Imaging was not performed in two patients: one had clinical progression, while the other discontinued treatment due to toxicity. Both patients were subsequently planned for best supportive care because of deterioration in performance status. The median time to response assessment imaging was 118 days. Before imaging, dose de-escalation to 200 mg was performed in 10 patients. Despite dose de-escalation, two patients had dose interruptions lasting more than 7 days; subsequently, they declined to continue pazopanib and were lost to follow-up. Among the 74 patients who tolerated 400 mg without dose de-escalation or treatment interruption lasting more than 7 days during the initial 4 months, only one patient required subsequent dose de-escalation and tolerated pazopanib at 200 mg until disease progression. Response rates and clinical outcomes are summarized in Table 2. The median follow-up was 41.3 months. The median PFS was 8.9 months (95% CI 6.7–11.0 months), and the median OS was 28.6 months (95% CI 19.0–38.1 months) (Figures 1 and 2). In an exploratory subgroup analysis, patients were stratified according to baseline ECOG performance status into Group A (ECOG PS 0–1; n = 46) and Group B (ECOG PS 2–3; n = 38). The median OS was 24.7 months in Group A and 40.9 months in Group B; however, the difference was not statistically significant (log-rank P = 0.603). Although OS was numerically longer in the ECOG PS 2–3 subgroup, this finding should be interpreted cautiously given the limited sample size and the exploratory nature of the analysis.

**Table 2:** Response rates and clinical outcomes (n = 84).

Response rates at first evaluation	Patient no. (%)
Complete Response	1 (1.2)
Partial Response	23 (27.4)
Stable disease	45 (53.5)
Progressive disease	13 (15.5)
Not evaluable	2 (2.4)
ORR	24 (28.6)
CBR	69 (82.1)
**Clinical outcomes**	
Median PFS	8.9 months
Median OS	28.6 months

CBR, clinical benefit rate; NA, not available; ORR, overall response rate; OS, overall survival; PFS,progression-freesurvival.

### Adverse event profile of reduced dose pazopanib

The median duration of exposure (without dose interruption) was 9.1 months. The most common adverse events were skin and hair depigmentation (35.7%), diarrhea (25%), hand–foot syndrome (21.4%) and hypertension (14.3%). There were no fatal treatment-related adverse events. No Grade ≥3 hematologic toxicity, arterial thromboembolic events, thrombotic microangiopathy, gastrointestinal perforation, interstitial lung disease, or cardiac events attributable to pazopanib were documented. Dose interruptions lasting more than 7 days were reported in 17 patients (20.2%). The toxicity profile is summarized in Table 3.

**Table 3:** Adverse effects (AE) of reduced-dose pazopanib.

Adverse events	Event number (%)
**Diarrhea**	
Grade 1 & 2	20 (23.8)
Grade ≥ 3	1 (1.2)
**Hand-foot syndrome**	
Grades 1 & 2	16 (19)
Grade ≥ 3	2 (2.4)
**Elevation of liver enzymes (Transaminitis)**	
Grades 1 & 2	6 (7.1)
Grade ≥ 3	1 (1.2)
**Hypertension (Including worsening)**	
Grades 1 & 2	11 (13.1)
Grade ≥ 3	1 (1.2)
**Fatigue**	
Grades 1 & 2	10 (11.9)
Grade ≥ 3	0 (0)
**Hyponatremia**	
Grades 1 & 2	9 (10.7)
Grade ≥3	0 (0)
Hypothyroidism	11 (13.1)
Skin & hair depigmentation	30 (35.7)
Altered taste (Grade not available)	8 (9.5)
Grade ≥3 adverse events related to pazopanib	5 (5.9)
Pazopanib-related treatment discontinuation	2 (2.4)
Dose de-escalation during treatment	11 (13.09)

### Post-progression treatment patterns

**Figure 3:**
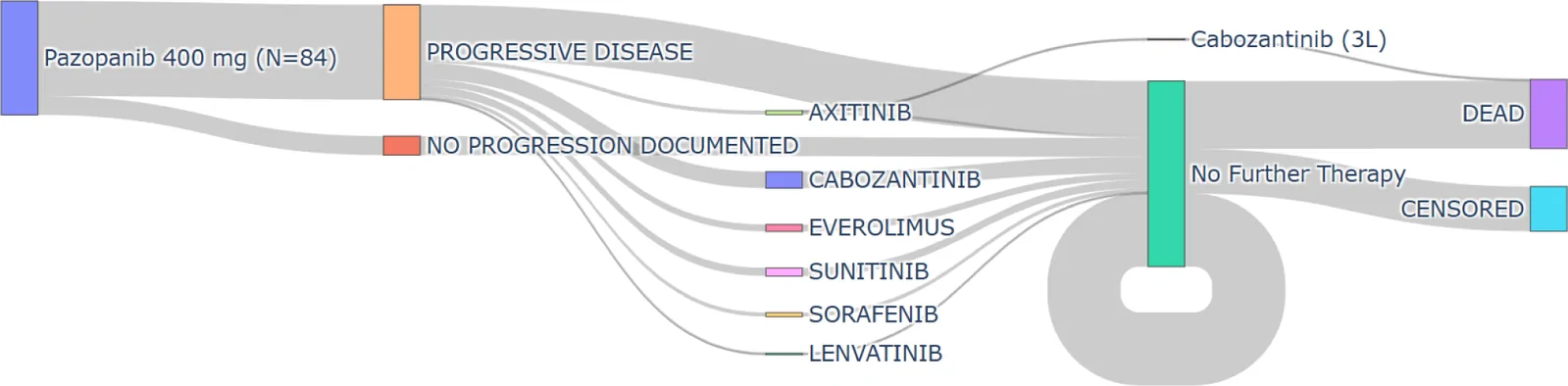
Sankey plot depicting treatment patterns post disease progression on pazopanib.

At the time of study completion, 70 patients (83.3%) had disease progression. All patients received counselling regarding next-line treatment options, expected survival outcomes, associated costs, and potential toxicities. However, only 30 of the 70 eligible patients (42.9%) received second-line systemic therapy after counselling. In more than three-quarters of patients, financial constraints were the predominant barrier to receiving second-line therapy. The most common second-line therapy was cabozantinib (n = 12). Only one patient received third-line therapy. None of the patients in our cohort received immunotherapy. The treatment patterns are illustrated in Figure 3.

## Discussion

In our study, reduced-dose pazopanib demonstrated clinically meaningful response rates and survival outcomes within the range reported in published studies of standard-dose pazopanib, while maintaining a favorable toxicity profile.

### Clinical and pathological characteristics

The clinicodemographic profile of our cohort was broadly comparable to that of previously published Indian kidney cancer datasets from Tata Memorial Hospital (12). Our cohort was younger (median age 57.5 years) than those in the Western real-world datasets, including the APOLAN study (69.6 years), PRINCIPAL study (66 years), and PAZOREAL cohort (69.7 years) (13, 17, 21). The study by Agnihotri et al. also demonstrated an earlier age of presentation in India, with a higher frequency of cases among quinquagenarian men (3). Nearly half of the patients in our cohort (45.1%) had an ECOG performance status ≥ 2, a population that was not represented in the landmark Sternberg et al. trial or the COMPARZ trial (8, 9). The lungs were the most common site of metastasis, and clear cell carcinoma was the most common histological subtype, similar to findings from the PARACHUTE study (22). However, a higher proportion of patients in our study had a metastatic burden involving more than two sites (36%) compared with the PARACHUTE study (26.9%) (22). This may be attributable to the late presentation of RCC in India, as described by Agnihotri et al. (3).

### Effectiveness of reduced dose pazopanib

Akatsuka et al. from Japan demonstrated that a low starting dose of 400 mg pazopanib did not affect clinical outcomes and resulted in better efficacy due to good tolerance. In our study, all patients received pazopanib (400 mg) as the initial dose. Our study had similar response rates when compared to the Japanese study in terms of ORR (28.6% vs 27.8%) and CBR (82.1% vs 83.3%). Median PFS were also comparable (8.9 vs 11.9 months) (16).

A retrospective study by Grassi et al. concluded that dose reduction of pazopanib significantly reduced the ORR (44% vs 19%) compared with the standard dose (800 mg). However, in the standard-dose cohort of this Italian study, only 3% of patients had an ECOG performance status ≥ 2, compared with 45.1% in our study (20). In the standard-dose cohort, 36% of patients required dose reduction during treatment, whereas in our study it was only 8.3%. In the Grassi et al. study, the median pazopanib exposure was shorter in the standard-dose group (800 mg) than in the reduced-dose group (9.6 months vs 13.6 months), and the former experienced a higher incidence of grade 3 fatigue (39% vs 6%) (20). Clearly, response rates (CR + PR) may be high with higher doses. However, in contrast to the European setting, where subsequent treatment options may be more readily available, in an LMIC setting where access to second-line treatment options is limited, the goal is to achieve a longer duration of disease control with comparable clinical benefit (CR + PR + SD) and less toxicity rather than to maximize response rates (23, 24). In this context, a reduced dose of pazopanib (400 mg) may be a feasible and clinically useful option, particularly for patients with poor performance status. In our study, more than one-fourth of patients (28.6%) achieved an objective response. The clinical benefit rate observed in our study cohort (82.1%) was comparable to that reported in landmark clinical trials and large real-world studies using the standard dose. These findings are depicted in Table 4.

**Table 4:** Comparison with standard-dose studies.

Variable	Our study	Sternberg et al. (8)	COMPARZ (9)	TMH study (11)	Roy et al. (4)	APOLON study (13)	PARACHUTE study (22)	PRINCIPAL study (17)	SPAZO (26)	PAZOREAL (21)
Number of patients	84	290	554	28	257	217	190	657	278	376
Dose	RDP	SDP	SDP	SDP	SDP	SDP	SDP	SDP	SDP	SDP
CR	1.2%	1%	<1	2.9%	1%	3.5%	3.1%	NA	4.6%	9.6%
PR	27.4%	30%	31%	19.6%	33%	45%	21.3%	NA	NA	NA
SD	53.5%	38%	NA	38.2%	47%	34%	44.1%	NA	NA	47.3%
PD	15.5%	18%	NA	20%	14%	NA	31.5%	NA	NA	NA
ORR	28.6%	31 %	NA	22.4%	34%	48.3%	24.4%	30.3%	30.3%	NA
CBR	82.1%	69%	NA	60.7%	81%	83%	68.5%	NA	NA	NA
Median PFS (months)	8.9	11.1	10.5	7.9	20.3	10.6	10	10.3	11.1	NA
Median OS (months)	28.6	NA	NA	12.8	22.7	29.1	NA	29.9	22.2	35.9

CBR, clinical benefit rate; CR, complete response; NA, not available; ORR, overall response rate; OS, overall survival; PFS, progression-free survival; PR, partial response; RDP, reduced-dose pazopanib; SD, stable disease; SDP, standard-dose pazopanib.

**Outcomes in patients with poor performance status:** Patients with ECOG PS ≥ 2 are generally underrepresented in prospective pazopanib studies, limiting the applicability of trial outcomes to this population. In our cohort, 38 patients (45.2%) had an ECOG PS 2–3, and exploratory analysis showed no significant difference in OS compared with patients with an ECOG PS of 0–1 (median OS 40.9 vs 24.7 months; log-rank P = 0.603). This numerical difference should be interpreted cautiously given the small sample size, retrospective design, and potential selection bias. The Pazo2 phase II study, which specifically evaluated pazopanib in carefully selected patients with ECOG PS 2, reported a median OS of 19.4 months, providing supportive evidence for the feasibility of pazopanib in selected patients with poor performance status (25). Notably, Pazo2 used the conventional pazopanib dose of 800 mg daily, whereas patients in our cohort received a reduced dose of 400 mg daily, with a median OS of 40.9 months among those with an ECOG PS of 2–3. Although direct cross-study comparisons are limited by differences in patient characteristics and study design, these findings are hypothesis-generating and raise the possibility that reduced-dose pazopanib may represent a pragmatic strategy for carefully selected patients with poor performance status, potentially improving treatment tolerability and treatment persistence while maintaining clinical benefit. On the other hand, although patients with an ECOG PS ≥ 2 were represented in other real-world cohorts, such as APOLON (17.6%) and SPAZO (approximately 19%), these studies did not report separate efficacy outcomes for this subgroup (13, 26). Thus, our cohort provides additional real-world evidence from a population with substantially greater representation of patients with an ECOG PS of 2–3. Prospective comparative studies are needed to determine whether reduced-dose pazopanib offers a clinically meaningful advantage over conventional dosing in this population.

### Safety profile of reduced-dose pazopanib

A major challenge in TKI therapy is avoiding treatment interruption and discontinuation due to treatment-related toxicity. The rate of permanent discontinuation due to pazopanib toxicity was significantly lower in our study cohort (2.4%) than in the PAZOREAL study (17.6%), in which all patients received pazopanib at a dose of 800 mg daily (21). Treatment discontinuation due to pazopanib toxicity occurred in 10.9% of patients in the PARACHUTE study, in which only 60% of patients received the full dose of 800 mg (22). Grade ≥ 3 adverse events may lead to treatment interruption or discontinuation and can adversely affect clinical outcomes. The rate of Grade ≥ 3 adverse events attributed to pazopanib was lower in our cohort than in the PAZOREAL study (5.9% vs 7.9%) (21). Although uncommon, fatal treatment-related toxicities were reported in the APOLON (2.7%), PAZOREAL (0.8%) and PARACHUTE (1.6%) cohorts (13, 21, 22). No fatal pazopanib-related toxicities were observed in our study.

The spectrum of adverse events observed in our study was similar to that reported in landmark studies of pazopanib (8, 9). However, the incidence of common toxicities, such as diarrhea (25% vs 52%) and hypertension (14.3% vs 40%), was less than half of that reported by Sternberg et al. (8). The incidence of hair color depigmentation was similar between the studies (35.7% vs 38%). Dose interruptions lasting more than a week were significantly less frequent in our study than in the COMPARZ trial (20.2% vs 44%) (9). In the PRINCIPAL and PARACHUTE studies, dose interruption rates were 40.5 and 51.7%, respectively (17, 22). Dose reductions to mitigate adverse events and to improve compliance were employed three times more frequently in the COMPARZ study than in our study (44% vs 13.1%) (9).

### Post-progression treatment patterns

Post-pazopanib treatment sequencing patterns observed in our cohort substantially varied from those reported in available clinical trials and real-world studies from developed countries. The proportion of patients receiving second-line therapy was significantly lower in our cohort than in the APOLON cohort (43% vs 70.7%) (13). When comparing post-pazopanib therapies between the APOLON and PARACHUTE studies, nivolumab was administered to 71.7 and 40.4% of patients, respectively, whereas none of the patients in our cohort received immunotherapy (13, 22). The second-line therapies received by patients in our cohort were less expensive treatment options, such as TKIs or mTOR inhibitors, which are considered inferior to the standard recommendation of immunotherapy. Financial constraints limiting access to immunotherapy among patients in LMIC were the main reason for this variation (7, 24). In the APOLON study, more than one-third (36.6%) of patients received at least two additional lines of systemic treatment after pazopanib, whereas only one patient (4.1%) in our study received third-line therapy (13). Patients’ inability to afford treatment and the lack of reimbursement for subsequent lines of therapy under government schemes resulted in many patients in our cohort being unable to receive further lines of treatment. Thus, in addition to the efficacy and tolerability of the treatment options selected, accessibility and affordability also influence treatment outcomes in LMICs (7, 24).

## Limitations

The major limitation of this study is its retrospective, single-center design. Evaluating the relevance of reduced-dose pazopanib in the era of immunotherapy through a clinical trial is not practically feasible. Moreover, the majority of our patients in our cohort would not meet the eligibility criteria of clinical trials. Underreporting of low-grade toxicities due to inherent limitations in documentation is another drawback, particularly given the retrospective nature of the study.

## Impact in LMIC Practice

To our knowledge, this represents one of the largest homogeneous single-center cohorts evaluating the effectiveness of reduced-dose pazopanib. Our patient cohort is underrepresented in clinical trials but reflects the profile of the majority of patients with metastatic kidney cancer in LMICs. Due to the unaffordability of immunotherapy and poor tolerance of standard-dose TKIs, many patients in resource-constrained settings receive reduced-dose pazopanib. In this context, high-quality real-world studies can help systematically document response rates and clinical outcomes associated with pragmatic reduced-dose treatment strategies. Despite the above-mentioned limitations, these real-world data from a tertiary cancer center in an LMIC setting offer a practically feasible treatment option that provides clinical benefit with fewer adverse events.

## Conclusions

Reduced-dose pazopanib is a feasible first-line treatment option for anti-VEGF TKI-naïve patients with borderline performance status who lack access to immunotherapy, offering clinically meaningful response rates and survival outcomes with a favorable toxicity profile.

## Informed Consent

This study was approved by the institutional review board (IRB). Vide no: 1616/IRB-SRC/13/MCC/13-12-2025/4. Being a retrospective study, waiver of consent was obtained from the IRB.

## References

[1] Padala SA, Barsouk A, Thandra KC, Saginala K, Mohammed A, Vakiti A, et al. Epidemiology of renal cell carcinoma. World J Oncol. 2020;11(3):79–87. 10.14740/wjon1279 32494314 PMC7239575

[2] Rose TL, Kim WY. Renal cell carcinoma: A review. JAMA. 2024;332(12):1001–10. 10.1001/jama.2024.12848 39196544 PMC11790279

[3] Agnihotri S, Kumar J, Jain M, Kapoor R, Mandhani A. Renal cell carcinoma in India demonstrates early age of onset and a late stage of presentation. Indian J Med Res. 2014;140(5):624–9. 25579143 PMC4311315

[4] Roy S, Biswas B, Dabkara D, Ganguly S, Ghosh J, Bhattacharjee A, et al. Demographic characteristics and treatment outcomes of advanced renal cell carcinoma with clear cell histology: A single-center experience from India. Cureus. 2024;16(6):e61978. 10.7759/cureus.61978 38855498 PMC11162510

[5] European Association of Urology. EAU Guidelines on Renal Cell Carcinoma 2026. Arnhem (The Netherlands): EAU Guidelines Office; 2026.

[6] So TH, Sharma S, Parij R, Spiteri C, Chawla E, Pandey P, et al. A systematic review to summarize treatment patterns, guidelines, and characteristics of patients with renal cell carcinoma in the Asia–Pacific region. Expert Rev Anticancer Ther. 2023;23(8): 853–63. 10.1080/14737140.2023.2236300 37458169

[7] Sahoo TP, Desai C, Agarwal S, Rauthan A, Dhabhar B, Biswas G, et al. ExPertConsEnsus on the management of Advanced clear-cell renal cell carcinoma: Indian perspective (PEARL-INDIA). BMC Cancer. 2023;23(1):737. 10.1186/s12885-023-11237-y 37558975 PMC10413514

[8] Sternberg CN, Davis ID, Mardiak J, Szczylik C, Lee E, Wagstaff J, et al. Pazopanib in locally advanced or metastatic renal cell carcinoma: Results of a randomized phase III trial. J Clin Oncol. 2010;28(6):1061–8. 10.1200/JCO.2009.23.9764 20100962

[9] Motzer RJ, Hutson TE, Cella D, Reeves J, Hawkins R, Guo J, et al. Pazopanib versus sunitinib in metastatic renal-cell carcinoma. N Engl J Med. 2013;369(8):722–31. 10.1056/NEJMoa1303989 23964934

[10] Deng H, Huang Y, Hong Z, Yuan X, Cao Z, Wei Y, et al. Pazopanib has equivalent anti-tumor effectiveness and lower total costs than sunitinib for treating metastatic or advanced renal cell carcinoma: A meta-analysis. BMC Cancer. 2019;19(1):489. 10.1186/s12885-019-5704-3 31122210 PMC6533682

[11] Guo J,Jin J, Oya M, Uemura H, Takahashi S, Tatsugami K, et al. Safety of pazopanib and sunitinib in treatment-naive patients with metastatic renal cell carcinoma: Asian versus non-Asian subgroup analysis of the COMPARZ trial. J Hematol Oncol. 2018 May 22;11(1):69. 10.1186/s13045-018-0617-1 29788981 PMC5964681

[12] Ramaswamy A, Joshi A, Noronha V, Patil VM, Kothari R, Sahu A, et al. Patterns of care and clinical outcomes in patients with metastatic renal cell carcinoma: Results from a tertiary cancer center in India. Clin Genitourin Cancer. 2017;15(3):e345–e355. 10.1016/j.clgc.2016.09.006 28077238

[13] Barthelemy P, Thiery-Vuillemin A, Lebret T, Bigot P, Stein U, Dourthe LM, et al. Real-world evidence in anti-VEGF naive patients treated with pazopanib for metastatic renal cell carcinoma (mRCC), the APOLON Study. ESMO Real World Data Digit Oncol. 2025;9:100155. 10.1016/j.esmorw.2025.100155 41646210 PMC12836544

[14] Tanaka H, Hiraga H, Takekuma Y, Harabayashi T, Nagamori S, Endo M, et al. Possibility for dose optimization of pazopanib from its plasma concentration in Japanese patients with cancer. Biol Pharm Bull. 2020;43(5):762–6. 10.1248/bpb.b19-00560 32115446

[15] Stitt TM, Saunders KM, Rowling BC, Waller JL, Clemmons AB. Reduced starting dose of tyrosine kinase inhibitors in patients with metastatic renal-cell carcinoma. J Hematol Oncol Pharm. 2022;12(3):138–44.

[16] Akatsuka J, Kimura G, Obayashi K, Tsutsumi K, Yanagi M, Endo Y, et al. Outcomes of starting low-dose pazopanib in patients with metastatic renal cell carcinoma who do not meet eligibility criteria for clinical trials. Urol Sci. 2021;32(3):104–10. 10.4103/UROS.UROS_145_20

[17] Schmidinger M, Bamias A, Procopio G, Hawkins R, Sanchez AR, Vázquez S, et al. Prospective observational study of pazopanib in patients with advanced renal cell carcinoma (PRINCIPAL Study). Oncologist. 2019;24(4):491–7. 10.1634/theoncologist.2018-0787 30867244 PMC6459236

[18] Sternberg CN, Motzer RJ, Hutson TE, Choueiri TK, Kollmannsberger C, Bjarnason GA, et al. COMPARZ post hoc analysis: Characterizing pazopanib responders with advanced renal cell carcinoma. Clin Genitourin Cancer. 2019;17(6): 425–435.e4. 10.1016/j.clgc.2019.01.015 31601514 PMC7518515

[19] Kyriacou NM, Gross AS, McLachlan AJ. Pharmacokinetics of pazopanib: A review of the determinants, influencing factors and the clinical importance of therapeutic drug monitoring. J Pharm Pharmacol. 2026;78(2):rgaf095. 10.1093/jpp/rgaf095 41451944

[20] Grassi P, Verzoni E, Ratta R, Porcu L, Prisciandaro M, Mennitto A, et al. Does dose modification affect efficacy of first-line pazopanib in metastatic renal cell carcinoma? Drugs R D. 2017;17(3):461–7. 10.1007/s40268-017-0203-y 28801802 PMC5629143

[21] Doehn C, Bögemann M, Grünwald V, Welslau M, Bedke J, Schostak M, et al. The non-interventional PAZOREAL study to assess the effectiveness and safety of pazopanib in a real-life setting: Reflecting a changing mRCC treatment landscape. Cancers (Basel). 2022;14(22):5486. 10.3390/cancers14225486 36428579 PMC9688275

[22] Erman M, Biswas B, Danchaivijitr P, Chen L, Wong YF, Hashem T, et al. Prospective observational study on pazopanib in patients treated for advanced or metastatic renal cell carcinoma in countries in Asia Pacific, North Africa, and Middle East regions: PARACHUTE study. BMC Cancer. 2021;21(1):1021. 10.1186/s12885-021-08738-z 34521387 PMC8442269

[23] Philip EJ, Zhang S, Tahir P, Kim D, Wright F, Bell A, et al. Cost-effectiveness of immunotherapy treatments for renal cell carcinoma: A systematic review. Kidney Cancer. 2021;5(1): 47–62. 10.3233/KCA-200107

[24] Gupta D, Singh A, Gupta N, Mehra N, Bahuguna P, Aggarwal V, et al. Cost-effectiveness of the first-line treatment options for metastatic renal cell carcinoma in India. JCO Glob Oncol. 2023;9:e2200246. 10.1200/GO.22.00246 36795991 PMC10166401

[25] Zarkar A, Pirrie S, Stubbs C, Hodgkins A, Farrugia D, Fife K, et al. A study of pazopanib safety and efficacy in patients with advanced clear cell renal cell carcinoma and ECOG performance status 2 (Pazo2): An open-label, multicentre, single-arm, phase II trial. *Clin Genitourin Cancer*. 2022;20(5):473–81. 10.1016/j.clgc.2022.06.01 35803859

[26] Pérez-Valderrama B, Arranz Arija JA, Rodríguez Sánchez A, et al. Validation of the International Metastatic Renal-Cell Carcinoma Database Consortium (IMDC) prognostic model for first-line pazopanib in metastatic renal carcinoma: The Spanish Oncologic Genitourinary Group (SOGUG) SPAZO study. Ann Oncol. 2016;27(4):706–11. 10.1093/annonc/mdv601 26658889

